# A Drone Logistic Model for Transporting the Complete Analytic Volume of a Large-Scale University Laboratory

**DOI:** 10.3390/ijerph18094580

**Published:** 2021-04-26

**Authors:** Karl-Arne Johannessen, Hans Comtet, Erik Fosse

**Affiliations:** 1 The Intervention Center, Oslo University Hospital, 0424 Oslo, Norway; hancom@ous-hf.no (H.C.); efosse@ous-hf.no (E.F.); 2 Faculty of Medicine, University of Oslo, 0318 Oslo, Norway; 3 The Department of Design, Norwegian University of Science and Technology, 7491 Trondheim, Norway

**Keywords:** drones, unmanned aerial vehicle, transport, health care, logistics

## Abstract

We present a model for drone transport of the complete annual analytic volume of 6.5 million analyses—(routine and emergency) between two inner-city university laboratories at Oslo University Hospital located 1.8 km apart and with a time restriction for the analyses of no more than 60 min. The total laboratory activity was analyzed per min for the complete year of 2018. The time from the clinical ordering of tests to the loading of the drone, drone transport time, and analysis time after the sample arrived at the analyzing laboratory were assessed using the lead time of emergency analyses of C-reactive protein, troponin, and the international normalized ratio. The activity had characteristic diurnal patterns, with the most intensive traffic between 8 and 12 a.m. on weekdays and there being considerably less traffic for the rest of the day, at night and on weekends. Drone schedules with departures 15–60 min apart were simulated. A maximum of 15 min between flights was required to meet the emergency demand for the analyses being completed within 60 min. The required drone weight capacity was below 3.5 kg at all times. In multiple simulations, the drone times were appropriate, whereas variations in the clinic- and laboratory-related time intervals caused violations of the allowed time 50% of the time. Drone transport with regular schedules may potentially improve the transport time compared with traditional ground transport and allow the merging of large laboratories, even when the demand for emergency analyses restricts the maximum transport time. Comprehensive economic evaluations and robust drone technology are needed before such solutions can be ready for implementation.

## 1. Introduction

Unmanned aircraft vehicles (UAVs, drones) are increasingly being adopted for transportation in a variety of services and are becoming part of health care transport. UAVs initially had military purposes; however, suggested civil applications include industrial surveillance, business parcel delivery, and imaging. Applications in the health sector, search and rescue following natural disasters, drug and vaccine delivery in rural districts, the provision of care technology in emergency situations, and the transportation of blood samples and organs have been topics of study [1,2,3,4,5,6,7,8].

The assessment of whether drone transport may be a sustainable future alternative for the highly scheduled transport of biological materials requires the study of real transport services to identify and overcome potential challenges that may arise under the conditions of strong wind and turbulence across infrastructure (i.e., around buildings and varying terrains) and large spans of temperature and precipitation as well as safety issues. Many authorities have implemented regulations relating to drone transport in the civil air space [9,10], and legislation regulating civilian drone flights with respect to safety, flight control and public tolerance is in place [11,12,13,14,15,16,17].

Multiple studies have adopted a conceptual framework of drone utilization based on tandem models and the traveling salesman problem to overcome the current limitations of flight distance and carrier weight in today’s drones [18,19,20,21,22,23]. However, with an increasing industrial interest in drones and the rapid development of drone technology, we foresee that combinations of different propulsive solutions, such as hydrogen fuel cells, electric batteries and solar energy, will extend the current range, time, and load limitations [24].

In the current study, we explore a logistic model for a potential full-scale drone solution to centralize and consolidate a large laboratory within Oslo University Hospital. In the case of inner-city transport between such large laboratories, short transport distances are less dependent on the limited drone range and may be handled through the frequent recharging of batteries. We therefore apply perspectives that are not restricted to the current drone range limitations, and we explore the feasibility and challenges of using a drone system for the transport of biological materials and blood products between the two largest sites of Oslo University Hospital.

There are several potential benefits for drone applications across larger laboratories. One potential benefit is that drone transport may enable the merger of large laboratories with duplicated services and infrastructure. Another is that a drone transport solution can outcompete existing ground transport. The aims of the current project were to investigate whether the complete analytic activity, carried out at the second largest laboratory of Oslo University hospital (located at Ulleval University Hospital), may be replaced by the drone transport of all laboratory specimens to the laboratory of the National Hospital for analysis, and to evaluate how such a solution may perform compared with the existing car transport.

We had two research aims:

1. Identify the crucial factors of a drone transport solution that may support a merger of two large hospital laboratories, and
2. Assess the time performance of such a model against the currently adopted ground transport system.

## 2. Background

### 2.1. Institution

Oslo University Hospital comprises four hospitals located within Oslo: the National Hospital (providing local, regional, and national services), Ulleval University Hospital (providing local, regional, and national services), Radiumhospitalet (a specialized cancer hospital) and Aker University Hospital (a local and central hospital). In 2018, Oslo University Hospital had total patient activity that included 94,000 hospitalizations, 45,000 day-care treatments and 853,000 outpatient consultations. The hospital had 24,000 employees, and patients were treated at more than 40 locations within a distance of 20 km. Oslo University Hospital is thus one of the largest hospitals in Europe and provides services that span from local hospital treatment to advanced specialized services and transplantations. With its complete range of medical services and large-scale economic and technical aspects, Oslo University Hospital covers multiple topics relevant to the assessment of UAV solutions as a complete service for the time-critical clinical transport of biological samples within large and complex institutions.

Our research was motivated by the fact that Oslo University Hospital is planning new buildings and a new structure to be established within 2030, with drone solutions being considered a future transport solution.

**Figure 1:**
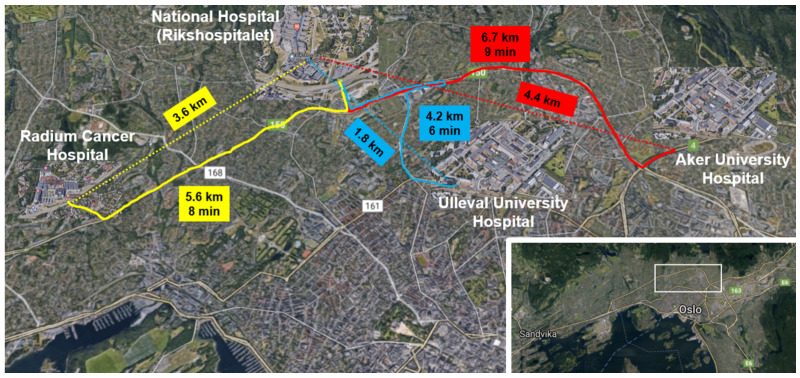
Hospital locations and distances. Lower-right corner: location in Oslo of the detailed section. Ground routes are labeled with distance and driving time, Straight lines show the Euclidean distances. Red lines: Aker Hospital-National Hospital. Blue lines: Ulleval Hospital-National Hospital. Yellow lines: Radiumhospitalet-National Hospital.

Although the Euclidean distance between the two laboratories that we focused on is 1.8 km (Figure 1), we anticipate that the drone may have to travel a longer distance owing to the dense residential area that surrounds the hospitals. On the basis of information provided by the Civil Aviation Authorities, we assume a flight distance of 3.6 km in our model.

### 2.2. Laboratory Services during the Study Period (2018)

Oslo University Hospital had laboratory services at all four main hospital locations; however, because the four laboratories did not conduct all types of analysis, critical transportation among the hospitals was needed. The total annual analytic volume at Oslo University Hospital in 2018 was close to 22.5 million analytic tests of 7.63 million biological samples (e.g., blood samples, biopsy specimens, and pathologic samples).

Point-of-care analyses (i.e., 3.2 million analyses performed in clinical wards) were not included in our analyses because such analyses were performed in the patient room/clinical ward and samples were not transported to the laboratories. At Ulleval University Hospital, laboratories conducted biochemistry, microbiology (i.e., bacteriology, virology, molecular diagnostics, and serology), and pathology analyses.

The analytic volume at Ulleval University Hospital in 2018 was 6.52 million laboratory analyses (2.059 million test samples), made up of analyses in the areas of clinical biochemistry (5.1 million laboratory analyses), microbiology (1.072 million laboratory analyses), and pharmacology (232,000 laboratory analyses) and 43,500 analyses of other laboratory services. Of the total analytic volume, 1.465 million (23.5%) of analyses were emergency analyses, and the ratio of hospitalized patient/outpatient analyses was 55.4%/44.5%.

### 2.3. Laboratory Costs in the Study Period

The current services organized at multiple locations incurred duplicated costs for infrastructure and personnel. For some laboratory specialties, this included 24/7 service with parallel teams of bioengineers and other staff.

The Division of Laboratory Medicine had a net area of infrastructure of 15,305 m^2^ at the National Hospital and approximately 12,708 m^2^ at Ulleval University hospital. In addition to the duplicated building structure, there was the servicing and maintenance of duplicated laboratory equipment. Because these two locations represent infrastructure of quite differing age (the National Hospital opened in 2000, whereas Ulleval University Hospital is considerably older), exact comparative costs of infrastructure do not exist. Some laboratories provided a service at only one location, whereas the Department of Medical Biochemistry was located at all four sites of Oslo University Hospital. Our study focuses on the National Hospital and Ulleval University Hospital, which were the largest units. The total costs and personnel costs suggest that appreciable savings can be made if these two laboratories merge (Table 1), and similar perspectives apply to Aker University Hospital and Radiumhospitalet.

A merger will not eliminate all duplicative costs at the hospitals, and it requires an upgrade of the resources at the National Hospital. From our new hospital project, we know that this is approximately 50%, indicating a cost reduction between EUR 10 and 20 million.

### 2.4. Ground Transport in the Study Period

The transportation of samples among the hospitals depended on road transport with three dedicated vehicles used in regular routing between 8 a.m. and 4 p.m. and taxis used the rest of the time and for urgent samples during the day. A major part of this transport between Ulleval University Hospital and the National Hospital was for routine analyses, but there were also emergency services. The transport time was at times unpredictable owing to traffic congestion and seasonal weather variations. No system was in place for the detailed control and monitoring of the transport.

The street between Ulleval University Hospital and the National hospital was partly a residential road, and partly a busy highway with heavy traffic in rush hour. The route had 15 crosswalks, each potentially delaying drivers for 15 s, and five traffic lights. Four of the traffic lights had waiting times of 15 s, and the fifth had a tram crossing that could cause a delay of 2.5 min on the route from the National Hospital to Ulleval University Hospital. The mean driving times without heavy traffic were 7.5 min from Ulleval University Hospital to the National Hospital and 9.2 min from the National Hospital to Ulleval University Hospital (via a different route). During rush hour in the morning, the route from Ulleval University Hospital could be delayed by traffic congestion, and in the afternoon, the route from the National Hospital could be similarly delayed in the opposite direction.

The variations in the timelines for regular car transport and taxi transport from Ulleval University Hospital to the National Hospital were obtained from the hospital annual service registry and are given in Table 2.

**Figure 2:**
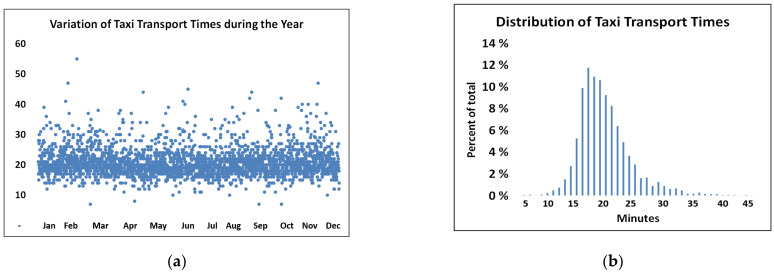
(**a**) Variation in transport times for 4800 taxi transports in 2018. (**b**) Distribution of the taxi transport times.

The minimal time of 27 min for routine car transport, as compared with the minimum possible time, suggests that the logistics of the routine transport are not optimal. The transport mode with the shortest transport time was by taxi, which was used only for emergency deliveries. The distribution of taxi transport times is shown in Figure 2. Although 90% of the taxi travel times are below 25 min, improvements might be made in the current solutions.

Figure 2 illustrates that a considerable proportion of the service times for emergency transport was well above the minimal transport time measured with no delays.

## 3. Clinical Units and Activities at Ulleval University Hospital

We established the demand profile of each clinic relating to planned and emergency services, the total activity at each of the five specialty laboratories, and the total transport demand of the complete laboratory activities.

The clinical activity was organized into 19 clinical divisions with 73 medical departments with a total of 230 (range of 1–24, mean of 4, and median of 4 units per department) medical subspecialty units (i.e., wards, outpatient clinics, and centers) providing a varying mix of inpatient treatments, day treatments, outpatient services, and emergency treatment.

A time restriction in our work was that emergency analyses should be completed within 60 min of the blood being drawn from the patient.

## 4. Methods

**Figure 3:**
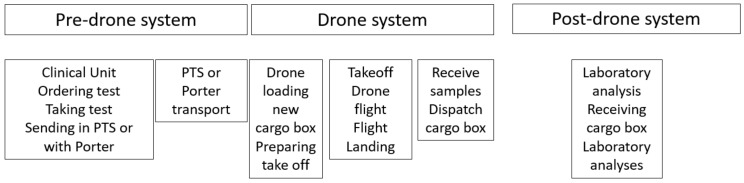
Structure of the analytical approach: pre-drone system including the clinical activity, drone system for transport from the clinic to the laboratory and post-drone system including laboratory analysis. (PTS = pneumatic transport system).

The macroscale perspective adopted in the analyses of our complete system is illustrated in Figure 3.

We used data for the pre-drone system in examining the overall time required for sample transport from the clinical unit hosting a patient to the laboratory receiving center (defined as the drone loading site in our model). This overall transport time comprised the clinical time C*_t_* (beginning with the clinical ordering of a blood test and ending with the sample dispatched by the pneumatic tube system (PTS) or a porter) and the transport time T*_t_* (beginning with the sample leaving the clinic and ending with the sample reaching the loading site). We included the whole repertoire of 463 analyses performed in 2018 in our volume analyses but used the times for tests of C-reactive protein (CRP), the international normalized ratio (INR) and troponin, routinely used for the benchmarking of time at our biochemical laboratory, when assessing transport lead times in our system.

For the post-drone system, we analyzed the laboratory time L*_t_*, beginning when a sample arrived at the laboratory reception center (assuming that the drone landing site was located there), ending when the analysis was completed in the laboratory.

**Figure 4:**
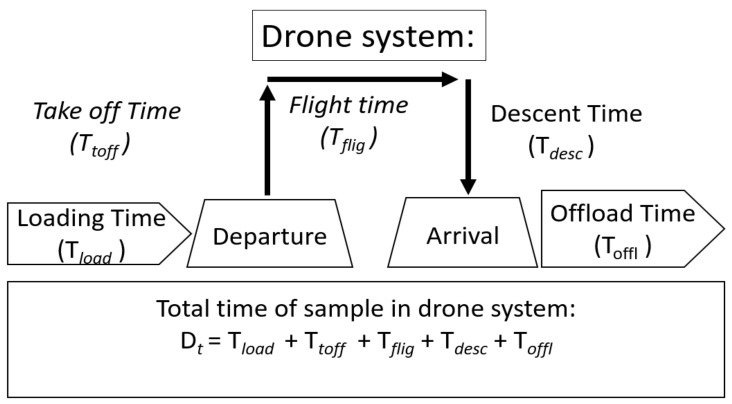
Model of the drone flight system and time sequence.

The macroscale perspective adopted in the analyses of our complete system is illustrated in Figure 4.

The drone loading time comprised the time needed for the drone to arrive at the loading station, the time needed to exchange an empty cargo box with a new loaded cargo box, and the time needed to prepare for takeoff. The flight time comprised the takeoff time needed to reach the flight altitude, the flight time at the set flight altitude, the descent time, and the landing time. Although the pre- and post-drone systems were extrinsic to the drone solution, the above measures defined the time margins for planning drone transport.

## 5. Analysis of Total Activity in 2018

The complete laboratory analytic activity in 2018 was analyzed with 1 min time resolution for a period of 365 days. Seasonal, monthly, weekly, daily, and diurnal patterns were mapped to analyze the required transport capacities and identify low-activity periods. We also targeted current clinical activity models that might have to be modified to reduce unnecessarily oversized drone capacities in periods of low activity.

The following analyses were performed:

1. Analysis of the total activity profiles across clinical units in characterizing the current patterns of transport volumes from all clinics to all five laboratories at Ulleval University Hospital.
2. Analysis of the mix of routine/emergency samples in evaluating the need for regular versus varying routing models.
3. Analysis of the time from the moment that a test was ordered in the clinical unit to the moment of the arrival of the sample at the drone loading site.
4. Analysis of the pneumatic tube system (PTS) and porter transport with particular focus on the transport time and arrival rate at the drone loading site.

### 5.1. Characterization of Transported Samples

**Figure 5:**
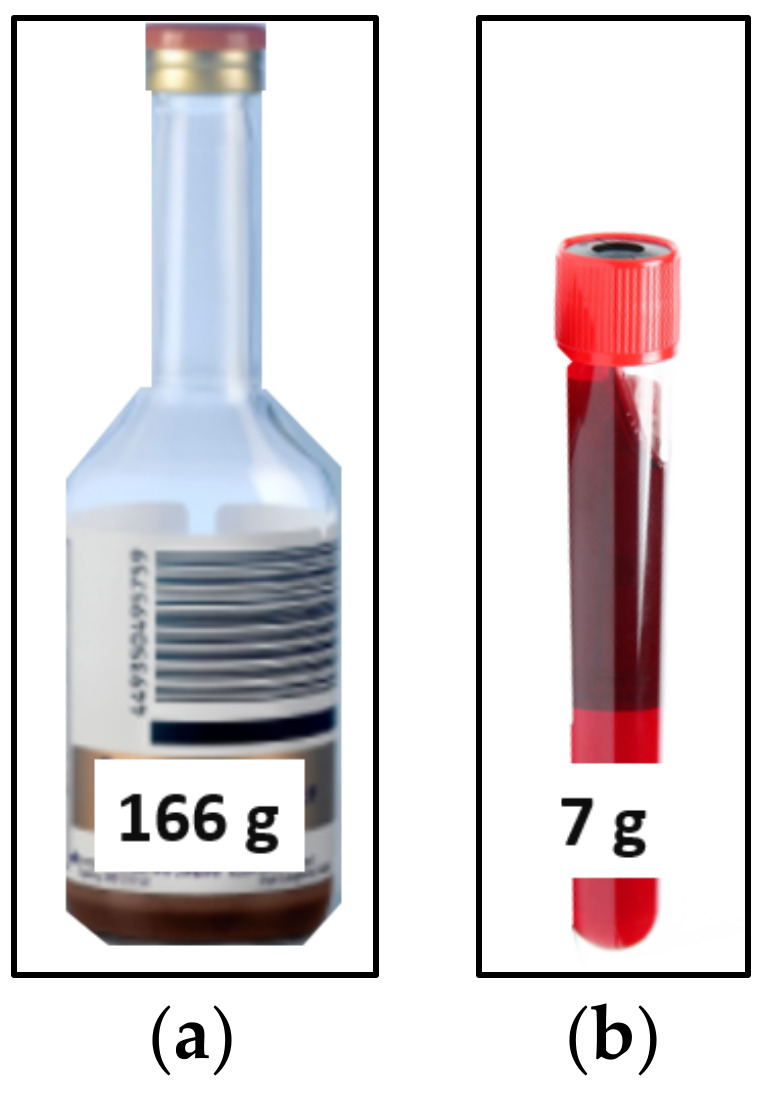
(**a**) Bottle weighting 166 g and having a volume of 50 mL; (**b**) Tube weighing 7 g and having a volume of 10 mL.

An important aspect was the varying weight and volume of samples arriving from different clinics. Our analyses quantitatively considered the number, weight, and volume of the sample specimens. Figure 5 illustrates how the test samples differed considerably in weight and volume. All test glasses were weighed assuming samples were filled as recommended by the manufacturer, not correcting for samples with smaller than the recommended volume. The weight per volume varied from 0.7 to 3.3 g/mL across the test samples. Other samples, such as those for urine tests, varied depending on the container used, and average was obtained based through manual weighing. The resulting weights were allocated to the ordered analyses, adjusting for the fact that multiple analyses may be made for one sample in a glass container. In some cases, an extra glass container was used in case of more analyses were needed, however, this was deemed to be of little consequence to the total weight (<1%).

### 5.2. Analysis of the Mix of Routine/Emergency Services

Our time limits were determined by the needs of emergency services. Certain clinics conducted more emergency activities than others, and we initially analyzed all data divided by planned and emergency services for the individual clinics and for the five individual laboratories. 

### 5.3. The Time from the Moment That the Blood Test Was Taken to the Moment of Arrival at the Drone Loading Site and Variation in the Time of PTS Transport to the Drone Loading Site

The time from clinical ordering to conducting a test was measured for the emergency samples because this was the decisive time in our model. We therefore used the current times relating to emergency analyses in our analyses of the drone system. 

### 5.4. PTS Transport and Arrival Times

Biological samples from different clinical locations were transported manually by porters or by the PTS to the loading site. We estimated the mean and maximum times of transport from all 67 PTS stations and the time of porter transport to the drone loading site for our simulations.

The electronic PTS monitoring system did not provide information on the number of biological samples in each vacuum tube, and we therefore assessed this number manually at the loading site on multiple days. The PTS transport times (PTS*_t_*) were analyzed for 28,000 transports using automatically recorded data of the electronic monitoring system, supplemented by the counting of 2000 samples in the tubes arriving at the drone loading site.

### 5.5. Post-Drone Times

The time required for the post-drone phase may vary as some analyses take longer to process than others (in terms of preparation and the time on instrument). We assessed the time required to process 50% of the samples and the time required to analyze 95% of the samples. 

## 6. Simplistic Approach Based on Queue Theory

Our PTS system might be considered a multi-server system because there were 67 PTS sender stations, each located at and dedicated to specific clinics and all sending samples to the common arrival station in the laboratory. However, as samples from these sender stations arrived independently of others at the loading site, as seen from the drone perspective, this uncoordinated current of samples from different clinics added up to an arrival pattern with a steady state that was within the acceptable limits.

Although this arrival pattern was considered an exogenic factor unrelated to the drone transport, we included this varying pattern in our evaluations of the drone capacity. We excluded periods in which the PTS system came to a complete halt for technical reasons and did not consider queuing models with abandonment, as observed for other systems, because all samples (equivalent to customers in other systems) had to remain in the system and not leave.

A well-known topic in queue theory is how customers prioritized in terms of wait times may be ranked according to principles such as first in–first out or first in–last out. These principles are aimed at reducing the waiting times for priority individuals (samples in our model) through earlier-arriving customers yielding to later-arriving customers. This was not relevant to our system because the first sample entering a drone always had the same departure time as the last sample entering (i.e., batching in a drone).

The PTS and drones in a sequence may also be considered a multi-server and multiphase system. However, although multiple drones might represent multiple servers, we planned a model where just one drone was available for loading at a given time. Assuming that a full transport box may be exchanged with an empty transport box without delay, we used a single queue–single server model because we intended to construct a simple model.

### Flight Time and Frequency in Drone Schedules

On the basis of the time limit of 60 min for emergency analyses, the allowable time for the drone system is given by:

(1) Drone system time D*_t_* = Emergency time restriction − C*_t_* − T*_t_* − L*_t_*.
  We therefore assessed guiding numbers for the pre- and post-drone time spans that were external to the drone system in our simulations. We also calculated the maximum time for the total system, which was our decisive variable for the planning of drone transport.
  The capacity of drone transport is a product of the number of drone flights per unit time multiplied by the load on each flight. The latter has a physical limitation in terms of the maximum drone weight or volume capacity, which must be considered (Figure 2).
  The total drone time D*_t_* is defined as the sum of the loading time (T*_load_*), take-off time (T*_toff_*), flight time (T*_flig_*), descent time (T*_desc_*) and offloading time (T*_offl_*):
  D_t_ = T*_load_* + T*_toff_* + T*_flig_* + T*_desc_* + T*_offl_*, where T*_toff_* + T*_flig_* + T*_desc_* = DFT,
  which is the air flight time of the drone (Figure 4).
  Each of the take-off time, flight time, and landing time may be affected by weather conditions and other air traffic. The flight height and path may depend on wind and turbulence and routing in relation to other traffic, and civil infrastructure may require the flight course to vary over time.
  Although the Euclidian distance was 1.8 km, we based our assumptions on a flight distance twice as long (i.e., 3.6 km), while the drone speed was set at 60 km/h, which was taken from a drone we used in preliminary tests (i.e., a Globe UAV Aquila Multicopter). This gave a value of 3.6 min for T*_flig_*. On the basis of multiple preliminary test flights, we assumed a period of 1 min for takeoff and a period of 3 min for landing. In our preliminary tests, the T*_flig_* was shorter with a tailwind and longer with a headwind; however, the total round-trip times were close to our assumed drone flight time (DFT) of 8 min.
  The loading time (T*_load_*) covers the time needed to get the drone in place for loading, the time needed to exchange a full transport compartment with an empty transport compartment and the time needed to prepare the drone for takeoff. The utilization of the drone capacity is a function of the filling of the drone, which depends on the arrival rate. T*_load_* is thus given in the next example.
(2) T*_load_* ≤ T_em_ − (Pre-drone time) − (Post-drone time) − DFT − T*_off_*.
  We assumed an offloading time T*_off_* = 0. T*_load_* is a possible limitation of the utilization of the drone capacity, as the extent of filling depends on the rate and allowed duration of filling.

## 7. Simulations

Across all the weeks in our analysis of laboratory activities, the maximum demand on any weekday at any time was ≤20% above the corresponding mean maximum for the same point in time. We therefore used a randomly varying increment of 20% in our simulations of the total system to assess the effects of such variations relative to the observed mean values across all variables and days in the period.

We used Excel (Microsoft Corporation, Redmond, WA, USA) and XLSTAT (Addinsoft Inc., Paris, France) for our simulations and statistical analyses.

## 8. Results

### 8.1. Overall Profiles across Clinical Units

Analyses of the activity in 2018 revealed large diurnal variations. The activity was highest during the morning on weekdays and substantially lower in the afternoons, at nights and on weekends. There were also large seasonal variations associated with holidays and vacations. The results from 8 weeks in March/April and 8 weeks in September/October, during which there were no vacations, were used in the analyses. All other time periods had lower volumes and were thus covered by the required capacity determined for the 16 weeks analyzed.

Compared with the biochemical laboratory, the other laboratories had rather small volumes and there was no need to consider emergency transport to the individual laboratories separately.

**Figure 6:**
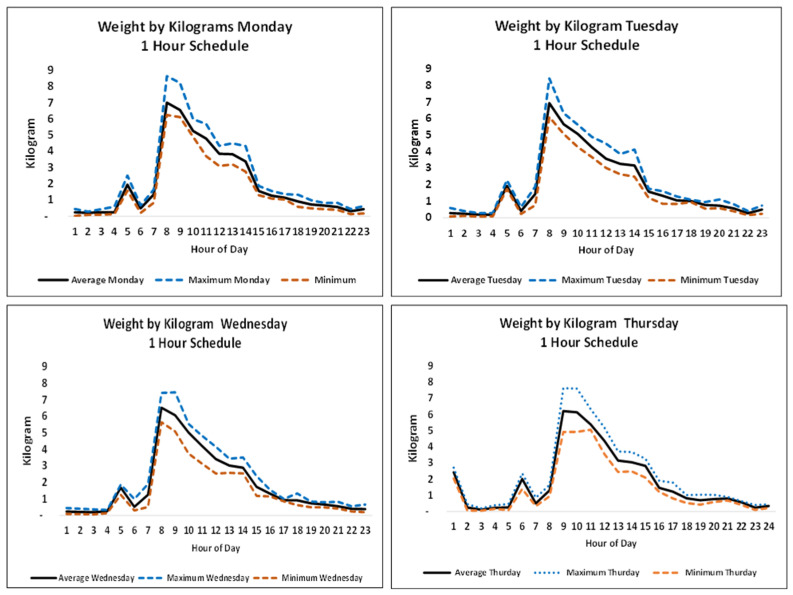
Average, maximum and minimum hourly payload weights on Monday–Thursday for 8 weeks of routine analyses at the medical biochemistry laboratory. Each maximum value was taken from the day with the highest value.

Figure 6 illustrates the typical routine activity per hour in the medical biochemistry laboratory on Monday–Thursday, the days having the highest activity. Similar profiles were observed for the other laboratories, however, with considerably lower peaks and volumes. Fridays and the weekends had lower activity and were easily covered by the schedule determined for the earlier days of the week. The figure shows time-varying activity with notable peaks throughout the daytime. The results for the schedule with a flight interval of 1 h suggest that the drone capacity can be downscaled during afternoons and nights, by either making fewer flights or using smaller drones. It is noted that morning activities required a drone with a loading capacity close to 8 kg.

### 8.2. Analysis of the Mix of Emergency/Routine Samples

**Figure 7:**
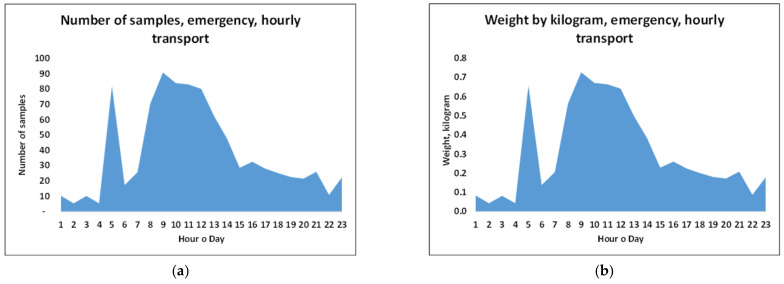

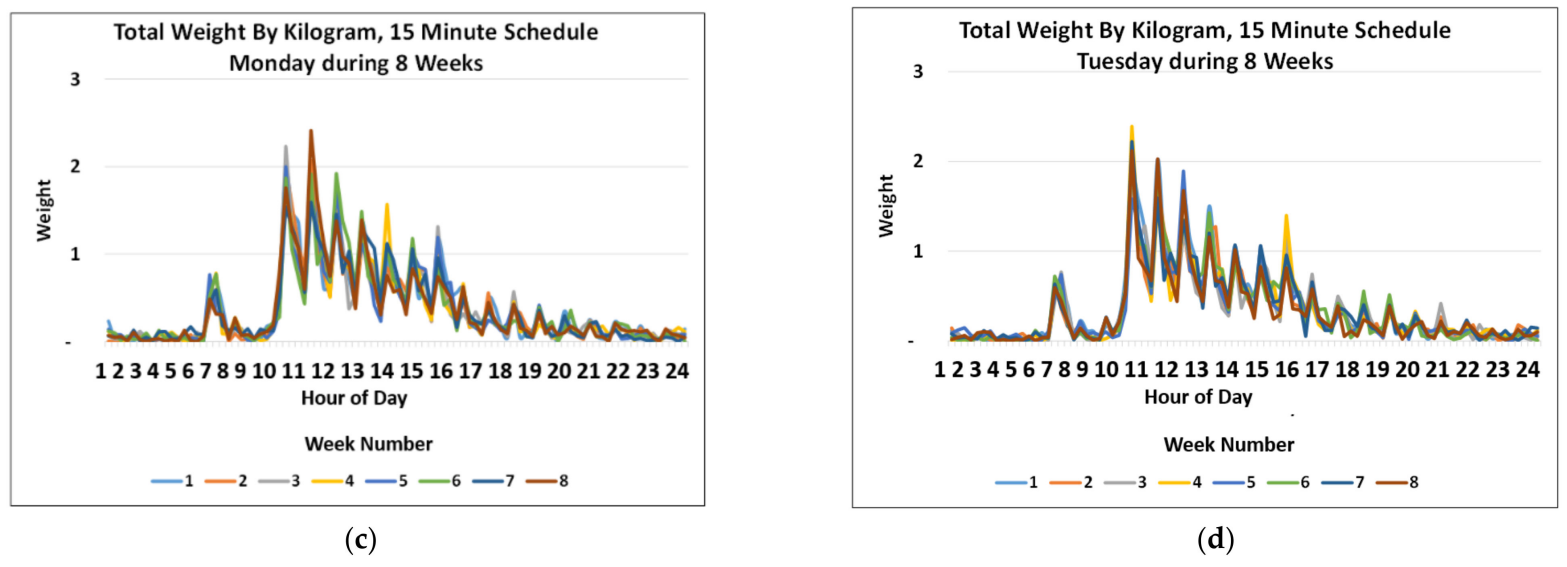
(**a**) Number of emergency laboratory samples. (**b**) Weight of samples corresponding to figure a. (**c**) Variation in the load weight on Mondays over 8 weeks for a 15 min frequency. (**d**) Variation in the load weight on Tuesdays over 8 weeks for a 15 min flight frequency.

The emergency activities of the medical biochemistry laboratory presented in Figure 7 reveal that schedules with an hourly frequency would not be sufficient. Although the volume of emergency samples was smaller volumes than the volumes of routine volumes, they constitute a continuous demand of time-critical services 24 h of the day. Accordingly, based on the urgency of emergency analyses, hourly drone flights would clearly not satisfy the time restriction of 60 min.

The non-drone times obtained in our analyses (Table 3) reveal that schedules with a 15 min frequency of drone departure are required. Figure 7c,d illustrates variations in the total weight on Mondays and Tuesdays during an 8 week period for a schedule with a 15 min flight frequency. The maximum deviations from the mean volumes at peak hour are ≤20%.

### 8.3. Pre-Drone System: Time from Clinic to Drone Loading Site

#### 8.3.1. Clinical Time

Although emergency analyses were of low volume compared with routine analyses, they decisively affect our system. The clinical time intervals of both routine and emergency analyses varied considerably; however, we assumed that the emergency analyses had the potential to have the shortest time intervals in the current operating situation. The main data of the emergency CRP, INR and troponin analyses are summarized in Table 3.

**Figure 8:**
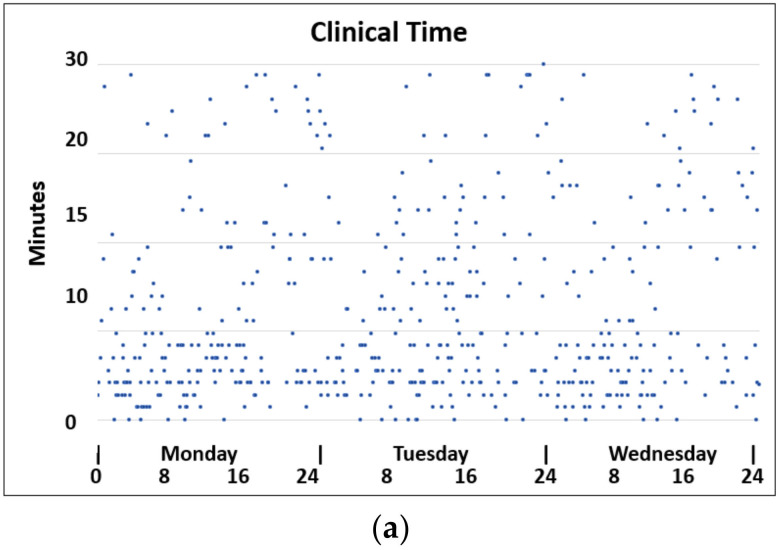

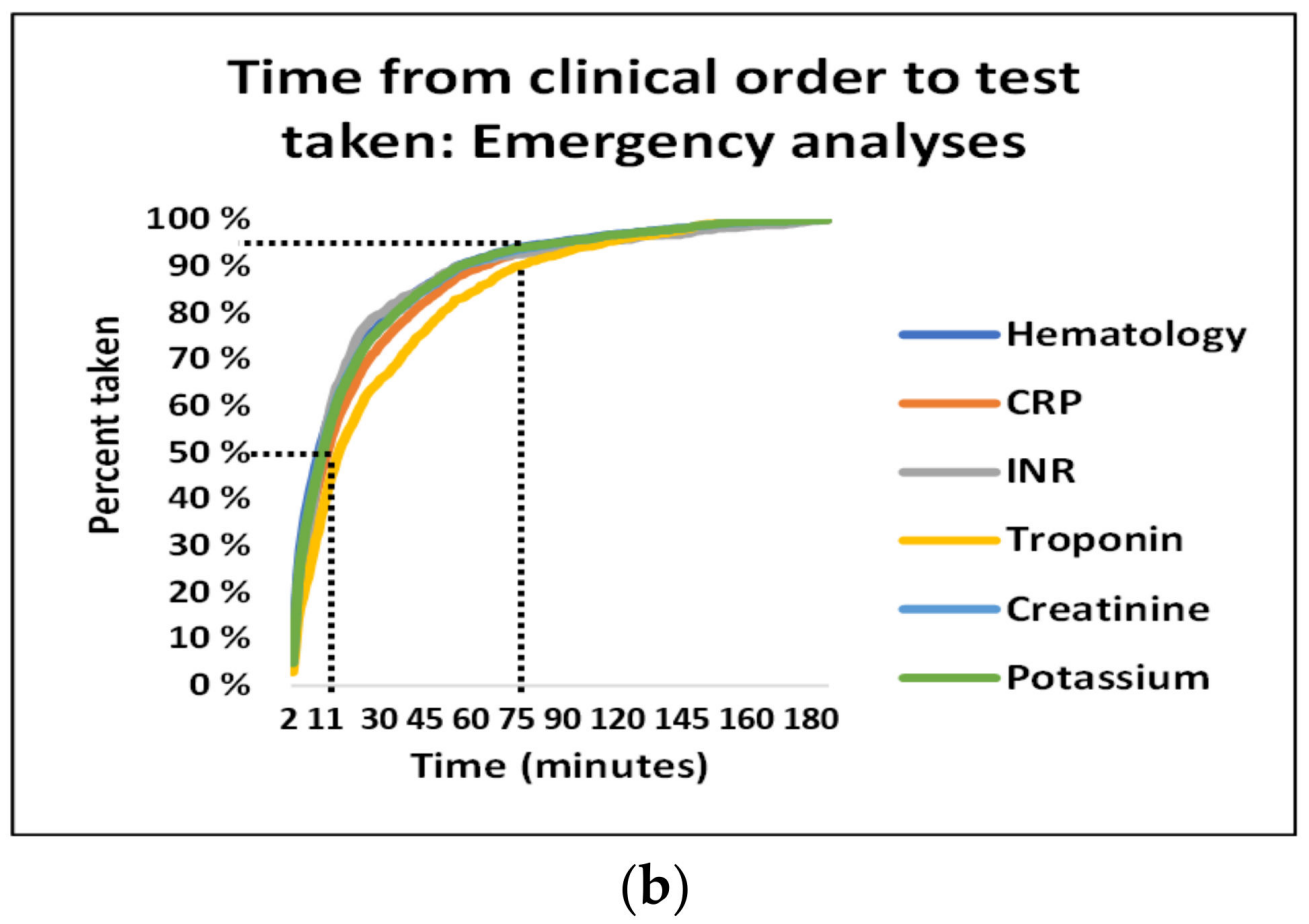
(**a**) Single time points. Diurnal measurements of clinical time. (**b**) Time from ordering to performing an emergency test.

Further characteristics of the pre-drone analyses are illustrated in Figure 8. The scatter plot in Figure 8a shows that some emergency samples were taken up to 30 min after being ordered. While 50% of the clinical orders of emergency samples were performed within 11 min, it took 75 min for 95% of clinical orders of emergency samples to be performed (Figure 8b). These time variations were not correlated to the typical periods of high activity but rather occurred during afternoons and at nights.

#### 8.3.2. PTS Transport Times

**Figure 9:**
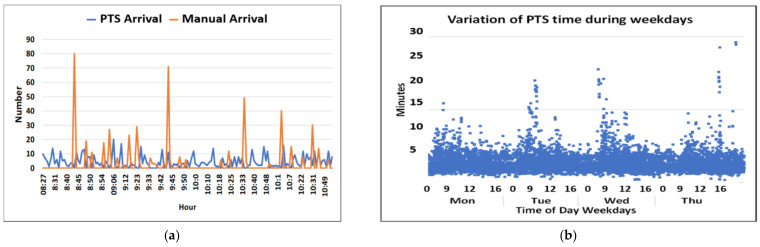

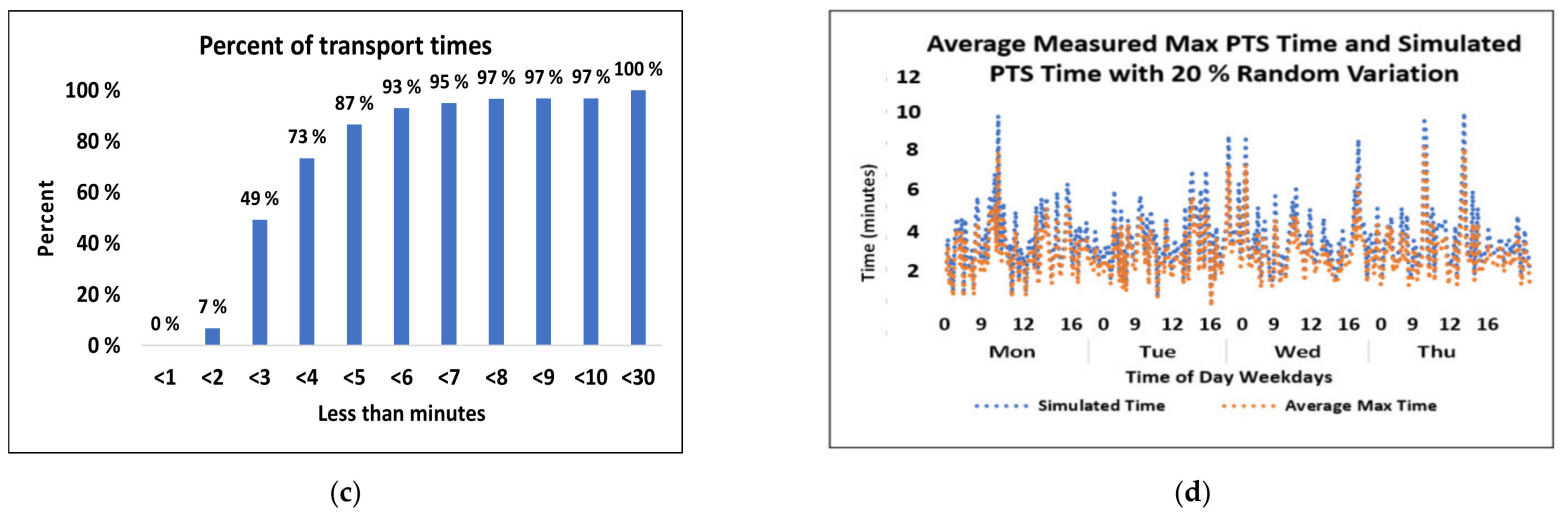
(**a**) Arrival rate measured at the drone loading site; (**b**) Measured transport time for an individual PTS; (**c**) Percentage of PTS times within time frames variation; (**d**) Simulated maximum PTS time allowing 20% random.

Figure 9 presents a typical time arrival curve during for the PTS and porter transport during busy morning hour. It is seen that the arrival rate per minute is determined by a combination of a random PTS process and the manual transport, which is a partly periodic and random process. The individual transport times for PTS tubes are depicted in panel b. Although the maximum transport time was 28 min on the selected days, panel c shows that 95% of transport times were within 7 min. Panel d compares the maximum transport time simulated using 20% random increases with the real measured PTS time. Such simulated maximum transport times were used in the simulation of the complete system.

### 8.4. Drone Filling Rate

Results of the drone filling rate are presented according to the arrival rates between 8 and 12 a.m. These were the busiest hour, which likely had the busiest filling rate, maximum capacity demand, and the maximum risk of queuing problems. Considering drone schedules with a flight frequency of 15 min, we assessed the filling of 3.5 kg drones in 15 min periods.

**Figure 10:**
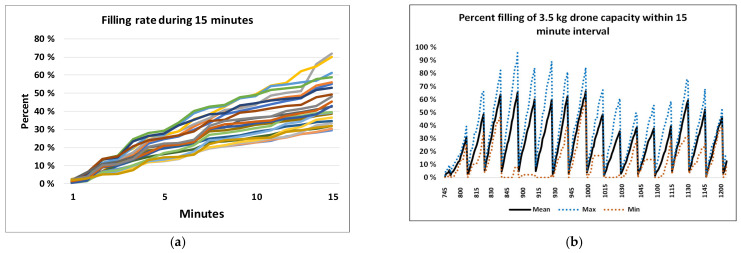
(**a**) Illustrations of the arrival and filling rates over 15 min intervals. Accumulated arrival of sample rates over 15 min intervals; (**b**) Variation in the percentage utilization of a 3.5 kg drone capacity.

Figure 10a shows the percentage filling for 20 filling periods of 15 min each, starting 5 min apart. The figure shows a variation in filling between 30% and 72% of the drone capacity. Figure 10b shows the corresponding percentage of accumulated filling of drones during 15 min periods starting 15 min apart. The figure confirms that the utilization of the drone varied with time.

### 8.5. Post-Drone System: Processing Time in the Laboratory

**Figure 11:**
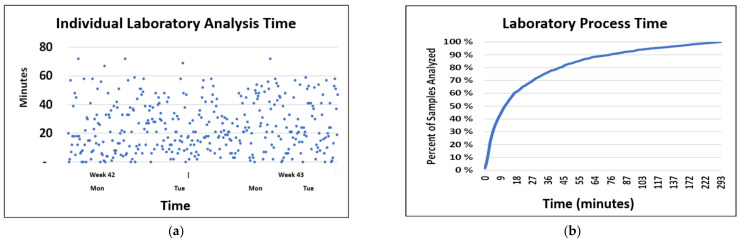
(**a**) Individual times of emergency analyses; (**b**) Accumulated times of emergency laboratory analyses.

The laboratory processing times were assessed for CRP, INR and troponin analyses as depicted in Figure 11. Although 50% of the analyses were processed within 15 min, the 95% percentile was not reached until 116 min.

## 9. Simulation of Predicted Full-Scale Transport Times

**Figure 12:**
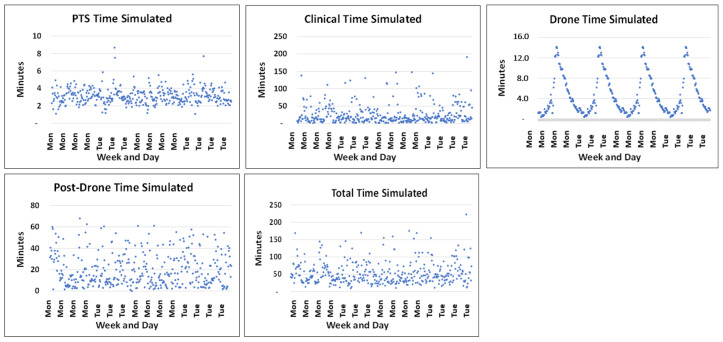
Individual times of PTS transport, clinical time, drone time, post-drone time and total time (sum of all) during simulations.

We simulated 10,000 events of the complete transport system allowing a random variation of arrival at the drone loading site and fixing the drone capacity at 3.5 kg. In contrast to the case for queuing systems using the average time in the service system, we used the maximum time in the system because no samples were intended to have a response time longer than 60 min. The total times for the system varied considerably as depicted in Figure 12 and Table 4.

Only 3% of the PTS times and none of the drone times were outside our intended time intervals. On average, 53% of the clinical times and 51% of the laboratory times were longer than 15 min and, thus, outside the assumed limits for these processes. Likely owing to the randomness of these measures, only 32% of the total times were outside maximum interval of 60 min.

## 10. Discussion

In the current study, we examined how time-varying clinical demands for laboratory analyses may affect future drone-based transport with the potential to merge a large laboratory with another. Our study showed that several factors must be considered in operating a large laboratory to avoid drone-related delays. Our model suggests that several gains relating to the economics and timeliness of services can be made. All the above are complex topics that require further study.

While the peak transport volumes per unit time determined the maximum needed drone load capacity during the peak h, the time restrictions imposed by emergency services determined the required frequency of the drone schedules 24 h a day. In our model with a university laboratory performing 6.5 million analyses per year, schedules with a 15 min flight frequency and drones with a load capacity of 3.5 kg could theoretically satisfied the demands of both the routine and emergency analyses. Although the time-critical emergency analyses were low in volumes and could easily be absorbed by a drone capacity of 3.5 kg, their urgency was a decisive criterion for the service.

We modelled a solution with a fixed drone capacity, representing a drone service that was oversized during evenings and holidays; i.e., that is, at times when there is less traffic. Further research is needed to assess the proper balance between the drone weight capacity, drone transport frequency, and routings from an economic perspective. This balance may depend on the local environment, the distance between institutions, other air traffic, the urban structures, and other factors.

Whereas a higher frequency of drone departures may reduce the required drone size, which would be favorable in urban regions where drones may be disruptive when flying through public spaces, a higher frequency of drone flights may also be disruptive and require more complex management of the air-space traffic. An important topic is whether solutions should be based on regular drone routings as in our model, on the transport demand, or on a combination of the two strategies. This may depend on both the mix of routine and emergency activities and the distances between locations. A topic that requires further research is whether on-demand drones should also be available for urgently required analyses.

Our analyses of the pre-drone–drone–post-drone system indicated that the drone service can be controlled using planned time intervals. This was related to the fact that the busiest times had rather small variations in volume, which were all covered by the estimated drone capacity, and that this peak capacity absorbed the required capacity of all other times of the day. Although we may not prevent many samples arriving at the same time (i.e., the drone load capacity may be exceeded), we consider this a low risk and that it will at most cause a 15 min delay as a sample waits for the next drone transport. The PTS system had variations outside the estimated limits in only 3% of the simulations, which was considered acceptable.

Our model indicates improvements in transport time over the currently used car transport services. However, optimized drone solutions should be compared with optimized ground transport. The suboptimal organization of ground transport is not an argument for using drones, and existing solutions should be studied and improved. Our observed car transport times indicate that the current system has potential for improvement, which should be established before strategic decisions are made. Furthermore, we assumed constant flight times in our model. Seasonal weather conditions may not only affect car transport as we experience today but also affect drone flight times. With current drone technology, it is not always possible for drones to fly at all. Improvements to the climatic sustainability of drones must be established before our model can be considered realistic to implement. Accordingly, conclusions of the benefit of drone services compared with ground transport must be made with care. Furthermore, not every service in health care is time critical, and economic evaluations must be made using appropriate standards to assess which time-related advantages of drone solutions will be sustainable compared with existing transport solutions.

Our model has a rather short distance between the two chosen laboratories involved. At larger distances, more time is needed for the drone flight than we used, which may affect the total response time and require higher drone frequencies and further optimization of the service [25]. However, we based our flight times on a drone speed of 60 km/h, and future drones may be able to fly considerably quicker [26,27], suggesting that potentially longer flight distances may be realistic in the future.

### Effect on the Clinical Organization

In contrast to the drone service performance in our model, both the pre-drone clinical time and analysis time during the post-drone time violated our allowable time intervals in 50% of simulations. Although the aggregated times exceeded the total time restriction in only 30% of simulations, such random totals would not be reliable. However, the corollary of the same fact is that almost 50% of the clinical and laboratory times were satisfactory, and lead times would be well within the required time limits if clinical routines were standardized. Accordingly, the lead times illustrate that our time restrictions may be realistic, given a clinical optimalization of logistics.

From a simplistic perspective, future drone solutions may be considered merely as a substitution of the current ground transport solutions with minimal consequences for clinical activities. In our model, however, improvements to the clinical logistics would be necessary to satisfy the defined time restrictions in our modelling. To what extent additional processing work should accompany the implementation of drone solutions may depend on local cultures and environments.

Tornatzky et al. [28] developed an interesting concept of the interplay among technology, the organizational culture and the environment, called the TOE framework. They categorized the innovation process into “developing” and “using”, portraying that the processes of innovation generation and adoption differ considerably. In some cultures, there may be an ambition to extend the implementation of new technologies to a broader innovative culture, whereas other cultures appear to mainly implement new technology by fitting it to existing solutions. Applied in a healthcare logistics setting, the TOE framework may offer factors affecting the decision to adopt technologies to improve healthcare logistics processes.

In our modelling, we identified necessary logistic improvements related to both the clinics and laboratory processes. Two well-known specialized methods from industry that focus on looking for waste, improving workflow and creating more value with less effort are the LEAN method (originating from the Toyota car manufacturing system) and Six Sigma (originating from Motorola). The experience of implementing such organizational processes varies from successful improvements to processes to cases in which implementing LEAN in clinical cultures has sparked further challenges that are more demanding than with the implementation of the methods in industry [29,30,31,32,33,34,35,36,37].

How to best engage clinical and laboratory leaders and managers in actively facilitating long-term improvement processes for the optimization of drone transport solutions requires extensive research. Approaches should be tailored to the existing organization and culture [38]. A new technology will invariably affect the process in which the technology is implemented, and processes often need to be aligned with the introduction of new technologies [39,40,41].

In our study, the outpatient analyses peaked at the same times of the day as hospitalized-patient analyses. Outpatient analyses constituted 44.5% of the total volume analyses, with the major activity occurring between 9 and 12 a.m. If the peak volume of the outpatient analyses had been 1–2 h later, the maximum transport capacities needed at this time would have been lower, possibly reducing the maximum needed drone weight capacity by 15%–20% and allowing the use us smaller drones. Whether such modifications would need comprehensive adjustments of clinical work schedules or by planning of only outpatient laboratory visits more flexibly, may be of interest in future studies.

We conclude that comparing drone transport with existing solutions, the logistics may require substantial refinement if the true potential of drone transport is to be achieved.

## 11. Sustainability of Future Drone Solutions

Most health care systems are battling increasing costs, and laboratory services with increasing complexity are crucial services that have escalating expenses with respect to infrastructure and operations on a daily 24 h basis. Drone transport may have the potential to reduce costs if, through its independence from ground traffic congestion and delays, it allows fast transport solutions that can centralize laboratory services that are traditionally performed at multiple locations with the duplication of infrastructure and 24/7 services.

If drones represent a transport system that offers close to 100% uptime with sufficient quality, they may contribute to the centralization of time-critical laboratory services, reducing both the operational costs and the costs of infrastructure investment. A recent report found that the quality of biological samples following drone turbulence is an important factor to consider [42]. Drone transport can be applied to other services, such as central sterile services providing operating instruments, hospital clothing, and hospital catering.

Although our drone modelling suggests that the costs of today’s duplicated services may be reduced by mergers or improved transport, this cost reduction cannot be outweighed by the costs relating to purchasing, maintaining, and operating of UAVs and the required launch infrastructures. The current economy of complex drone transport is not known in detail sufficient for complete economic evaluation; however, studies have been published [8,43], and the history of technological development has demonstrated that the costs of technological innovations decline appreciably over time [44,45]. We did not focus on solutions that might minimize the total cost of transportation. Economically efficient solutions may require drone fleets to meet the maximum capacity needs and time constraints of the medical demands as well as to adapt to diurnal and seasonal variations in the required capacity.

We targeted the use of an uncomplicated model in our system. Our goal was to portray a drone service model with adequate but not excessive capacity, where the key trade-off is between the cost of excess capacity in low-demand periods and the risk of a service that is insufficient in high-demand periods. We have no knowledge with which to conclude what would be the best solution, and more research is required. One option that should be considered for future time-varying drone solutions, as in our modelling, is whether the vacant drone capacity in less busy periods may be used for other purposes.

The adoption of point-of-care testing has increased in the past few years because it reduces the turnaround time [46,47,48]. Future laboratory activities might include such point-of-care analyses, centralized laboratories (core laboratories) conducting 24/7 activities and dedicated laboratories conducting specialized analyses having demands that are less time critical. This may in turn reduce the needed biological transport volumes for external inter-institutional transport, contributing to both simpler solutions and lower capacity demands. When assessing future sustainability, lower transport needs for future transport than needs observed today should be expected.

## 12. Limitations

Although our modelling is based on comprehensive data and reflects large laboratories providing a mixture of routine and emergency services, it is related to a structure having a Euclidean distance of only 1.8 km. Other hospital systems may have longer distances. We foresee that longer flight distances will apply to urban hospital locations. Inner-city transport may be relevant across multiple types of laboratories, for which future solutions are emerging [49,50].

A stable real-life drone service may depend on several extrinsic factors, such as the weather, other air traffic and landing sites. A longer flight distance may result in higher risks of variation in the flight times, variation in regularity of services as well as delays. Undoubtedly, strong wind conditions may prolong the flight time, and such topics are part of our current research [51]. Furthermore, the tolerance of biological materials to flight conditions, such as turbulence and temperature, is an important consideration [6,42].

We chose to apply a simple drone routing. More sophisticated models may have advantages from both economic and service perspectives, and research on more complex solutions is needed. In particular, we believe that flexible flight routings should be explored.

We assumed a fixed landing site in our modelling, setting the site for landing and drone loading at the reception center of the current laboratory. More complex institutions may require multiple service locations and models to operate multiple landing sites. This may involve more complexity with respect to both the planning of drone routing and economics.

## 13. Conclusions

We conclude that drone transport models offer transport solutions for large-scale laboratory services with the potential to improve service time and laboratory costs, but that such gains depend on multiple factors that require further investigation.

Drone transport may enable the merger of duplicated services with reduced costs of service and infrastructure. Increased knowledge of drone service costs, including the costs of operation, infrastructure, and maintenance are needed, and the effects of local demands and the framework may be crucial.

Comparisons of the time gains between traditional logistics and drone services require that the compared solutions be optimized before conclusions are made, and the final conclusions may depend on the distance, climatic conditions, geographical location, and type of institution. Importantly, weather conditions which today pose a challenge to ground transport, are a great threat to the stability of drone traffic.

Care should be taken in considering any health care related service as time critical. Those services that really are time critical require regular and stable drone services, which will not be realistic until drones are more robust against physical conditions.

## Figures and Tables

**Table 1:** Total costs of all laboratory services in the Division of Laboratory Medicine and the costs related to the Department of Medical Biochemistry for 2018. The Department of Medical Biochemistry is organized at four locations. (Annual costs in million Euro. Average exchange rate 2018 NOK/€ = 9.4).

Organizational Unit	Total Costs	Personnel Costs
Division of Laboratory Medicine	256.8	184.9
Department of Medical Biochemistry (MBC)	44.3	27.3
MBC National Hospital	10.1	7.4
MBC Ulleval Hospital	9.6	7.7
MBC Other Locations	24.6	12.2

**Table 2:** Calculated minimum driving time from Ulleval University Hospital to the National Hospital with no delays. The minimal and maximum observed times are the actual times registered in our databases.

Current Ground Transport Times (Minutes)			
Transport Type	Calculated Minimum	Minimal Observed	Maximum Observed
Routine transport	7.5 min	27 min	170 min
Taxi transport	7.5 min	7 min	55 min

**Table 3:** The time elements in the transport model.

Time Measures for Emergency Analyses (minutes)				
	Mean	SD	Min	Max
Clinical Time	3.5	3.2	1.0	180
PTS transport	3.2	1.4	0.1	28
Laboratory Process Time	28.1	10.5	9.1	125
Total Non-Drone Time	34.7	15.1	9.1	333

**Table 4:** Percent of simulations violating the maximum allowed total time of 60 min.

Time Measure	Mean	SD	Min	Max
Total Time in System	32%	0.9%	30%	33%
% PTS > 5 min	3%	0.7%	2%	4%
% Clinical times > 15 min	53%	0.7%	52%	55%
% Laboratory time > 15 min	51%	0.8%	49%	53%
% Drone Times > 15 min	0%	0%	0%	0%

## Data Availability

Our data are not available for general distribution. Please consult corresponding author for further information if interest.
